# Leptin Counteracts the Hypoxia-Induced Inhibition of Spontaneously Firing Hippocampal Neurons: A Microelectrode Array Study

**DOI:** 10.1371/journal.pone.0041530

**Published:** 2012-07-25

**Authors:** Daniela Gavello, Jonathan Rojo-Ruiz, Andrea Marcantoni, Claudio Franchino, Emilio Carbone, Valentina Carabelli

**Affiliations:** Department of Drug Science and Technology, NIS Center, CNISM, University of Torino, Torino, Italy; University of Bristol, United Kingdom

## Abstract

Besides regulating energy balance and reducing body-weight, the adipokine leptin has been recently shown to be neuroprotective and antiapoptotic by promoting neuronal survival after excitotoxic and oxidative insults. Here, we investigated the firing properties of mouse hippocampal neurons and the effects of leptin pretreatment on hypoxic damage (2 hours, 3% O_2_). Experiments were carried out by means of the microelectrode array (MEA) technology, monitoring hippocampal neurons activity from 11 to 18 days in vitro (DIV). Under normoxic conditions, hippocampal neurons were spontaneously firing, either with prevailing isolated and randomly distributed spikes (11 DIV), or with patterns characterized by synchronized bursts (18 DIV). Exposure to hypoxia severely impaired the spontaneous activity of hippocampal neurons, reducing their firing frequency by 54% and 69%, at 11 and 18 DIV respectively, and synchronized their firing activity. Pretreatment with 50 nM leptin reduced the firing frequency of normoxic neurons and contrasted the hypoxia-induced depressive action, either by limiting the firing frequency reduction (at both ages) or by increasing it to 126% (in younger neurons). In order to find out whether leptin exerts its effect by activating large conductance Ca^2+^-activated K^+^ channels (BK), as shown on rat hippocampal neurons, we applied the BK channel blocker paxilline (1 µM). Our data show that paxilline reversed the effects of leptin, both on normoxic and hypoxic neurons, suggesting that the adipokine counteracts hypoxia through BK channels activation in mouse hippocampal neurons.

## Introduction

Leptin is an adipokine produced by fat tissue and encoded by the *Ob* (obese) *gene*. It is characterized by a receptor-mediated action exerted through a class I cytokine receptor [1] associated with JAKs (Janus Tyrosine Kinases) and it activates a signalling cascade mediated by PI3K (phosphoinositide 3-kinase) and the pathway of Ras-Raf-MAPK [2], [3]. Besides its body-weight reducing effects [4], increasing evidence has recently shown that leptin may reduce neuronal activity [5] and may promote neuronal survival from different injuries, such as excitotoxic and oxidative insults [6]. Leptin has also clear therapeutical effects, as demonstrated by its ability to reverse the loss of dopaminergic neurons in a model of Parkinson’s disease [7]. Moreover, leptin exerts a neuroprotective action against ischemic injury in rat cortical and hippocampal neurons, reducing neuronal cell death [8], [3]. There is also evidence for a role of leptin in opposing cell death mechanisms, through a ERK1/2, MAPK and STAT3 signalling pathway [9], [3].

**Figure 1:**
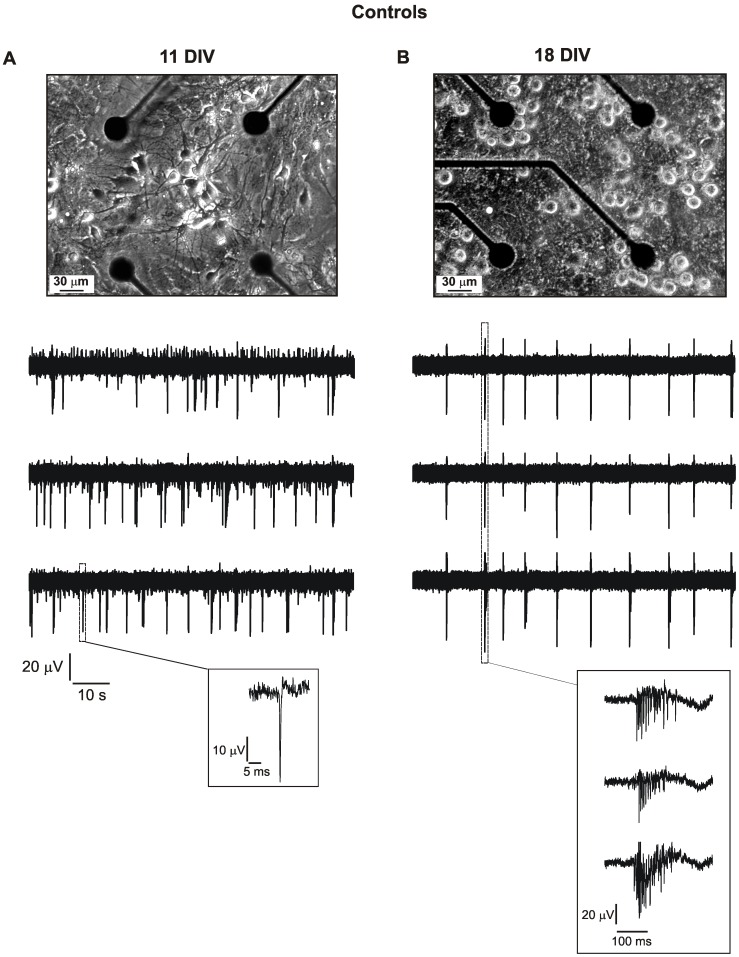
Spontaneous activity of hippocampal primary cultures at different ages. **A.** Photograph of an hippocampal primary culture on DIV 11, plated over an array of planar microelectrodes (top). The photograph is a detail relative to 4 over the 60 recordings electrodes of the MEA. Representative spontaneous electrical activity recorded from three different channels of the same MEA, on DIV 11 (bottom). In the inset is visible a magnification of a single extracellularly recorded action potential, since single spike firing is the characterizing feature of young cultures. **B.** Photograph of an hippocampal primary culture on DIV 18, plated over an array of planar multi-electrodes (top). Examples of spontaneous electrical activity recorded from three different channels of the same MEA, on DIV 18 (bottom). Inset: magnification of a burst, the typical pattern of activity in elder cultures.

In the past ten years, several works pointed out the correlation between hypoxia and the increased risk of AD (Alzheimer’s Disease): a period of reduced oxygen supply, occurring during chronic cerebral hypoperfusion (CCH) or cerebral ischemia, which may lead to the up-regulation of β-amyloid precursor protein cleavage enzyme 1 (BACE-1) and downstream neurotoxic plaques formation [10], [11]. So far, most studies on leptin’s neuroprotective effects were performed on the leptin-mediated neuronal survival by means of cell viability assays, while only few reports focused on the effects on neuronal activity [12]. A functional characterization of neuronal firing properties under normoxic and hypoxic conditions would be of great interest in this regard, since malfunctioning in signal transduction has been shown to be related to the first symptoms of neurodegenerative disorders, while cell death occurs at later stages of the disease [13].

In this work we investigated this critical issue by taking advantage of the MEA devices, which allow repeated non-invasive multisite recordings at different days in vitro [14], [15]. We compared the firing properties of mouse hippocampal neurons at 11 and 18 DIV, and monitored the effects of leptin, both under normoxic and hypoxic conditions. Our data demonstrate that spontaneous firing at 11 and 18 DIV is severely compromised by hypoxia, although at a different extent (54% versus 69% reduction, respectively). Leptin, on its own, exerts different effects on normoxic and hypoxic neurons. It significantly decreases the firing frequency in normoxic neurons but, when applied during hypoxia, leptin either counteracts the hypoxia-induced reduction of spontaneous activity or even potentiates the firing frequency of 11 DIV hippocampal neurons. Thus, even if with different effects on younger and elder neurons, overall leptin reverses the inhibitory action of hypoxia on the firing properties of hippocampal neurons. On the contrary, application of leptin together with the BK channel blocker paxilline (1 µM), prevents leptin action and restores the hypoxia-induced reduction of spontaneous firing.

## Materials and Methods

### Cell Culture on MEAs

All experiments were performed in accordance with the guidelines established by the National Council on Animal Care and approved by the local Animal Care Committee of Turin University. Hippocampal neurons were obtained from black-six mouse 18-day embryos. Hippocampus was rapidly dissected under sterile conditions, kept in cold HBSS (4°C) with high glucose, and then digested with papain (0,5 mg/ml) dissolved in HBSS plus DNAse (0,1 mg/ml) [16], [17]. Isolated cells were then plated at the final density of 1200 cells/mm^2^ [18] onto the MEA (previously coated with poly-DL-lysine and laminine) and allowed to adhere on the centre of the chip by using a ring made of Sylgard 184 (Dow Corning), 4.5 mm internal diameter (11 mm external diameter), which was removed after 4 hours. The cells were incubated with 1% penicillin/streptomycin, 1% glutamax, 2.5% fetal bovine serum, 2% B-27 supplemented neurobasal medium in a humidified 5% CO_2_ atmosphere at 37°C. Each MEA dish was covered with a fluorinated ethylene-propylene membrane (ALA scientific, Westbury, NY, USA) to reduce medium evaporation and maintain sterility, thus allowing repeated recordings from the same chip. Recordings were carried out since DIV 11 until DIV 18. Culture medium was partially (1/3) changed once a week, depending on the age of the culture (young cultures did not need weekly change of medium). Following experiments, MEA dishes were re-used by cleaning overnight in 1% Tergazyme (Sigma-Aldrich, St. Louis, MO), rinsing in distilled water and then sterilizing overnight under UV ray.

**Figure 2:**
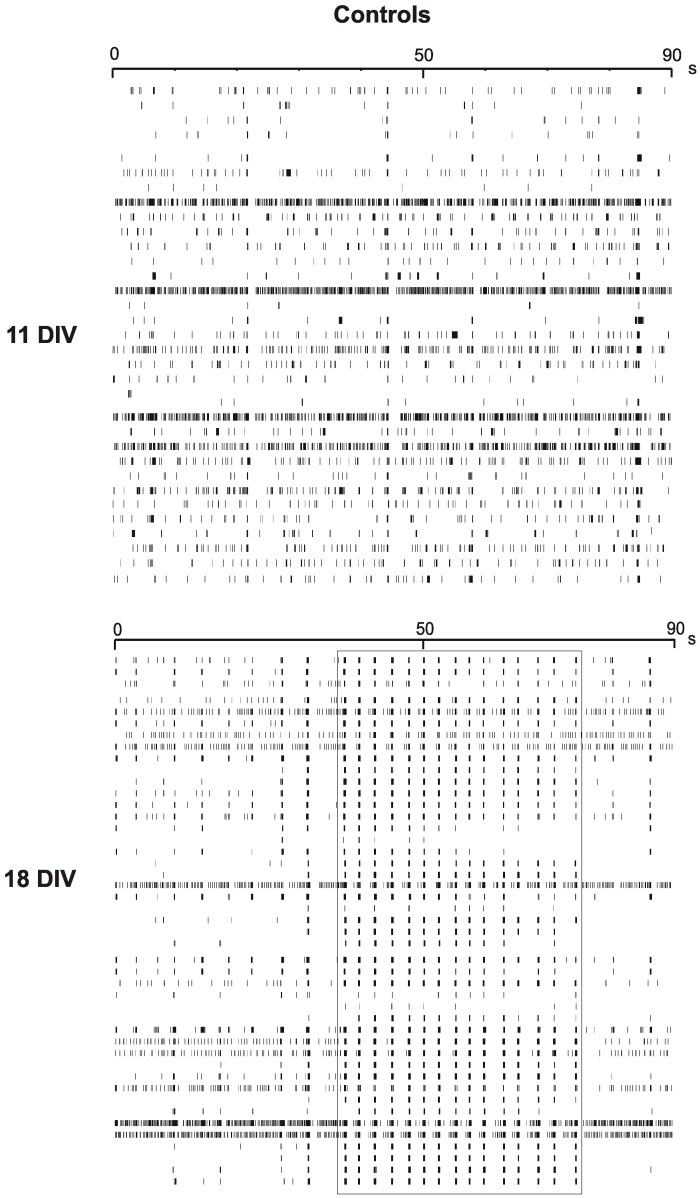
Network activity changes along with culture development. Raster plots of two representative MEAs at DIV 11 (top) and DIV 18 (bottom). Each line represents the signals detected by a single electrode of the MEA array, during 90 seconds recordings. Uniquely active channels are shown. Synchronization of the spontaneous activity is almost absent in younger cultures, while it is clearly visible at DIV 18 (rectangle).

### MEA Recordings

Multisite extracellular recordings were performed using the MEA-system, purchased from Multi-Channel Systems (Reutlingen, Germany). The 60 electrodes array (TiN/SiN) is composed by a 8×8 square grid with 200 µm inter-electrode spacing and 30 µm electrode diameter. Data acquisition was controlled through MC_Rack software (Multi-Channel Systems Reutlingen, Germany), setting the threshold for spike detection at −15 µV and sampling at 10 kHz. Experiments were performed in a non-humidified incubator at 37°C and with 5% CO_2,_ without replacing the culture medium.

Before starting the experiments, cells were allowed to stabilize in the non-humified incubator for 90 seconds; then recordings of the spontaneous activity was carried out for 6 minutes.

For hypoxic treatment, cells were kept for 2 hours into a humified incubator with 5% CO_2_ and 3% O_2_ (37°C); after that, spontaneous activity was measured under normoxic conditions. The recovery was recorded 2 hours after hypoxia.

Murine recombinant leptin (PeproTech, London, UK) was used at a final concentration of 50 nM, being applied 1 hour before the recordings, under hypoxic or normoxic conditions, and maintained in the MEA dish for the whole recording. Then it was washed out and we waited 2 hours before recording the recovery. Paxilline (Sigma-Aldrich, St. Louis, MO) was dissolved in DMSO (Sigma-Aldrich, St. Louis, MO) and used at the final concentration of 1 µM [19]. Cultures were kept into the normoxic incubator between recording sessions. It is worth noticing that, due to the variability among different MEAs, the effects of any treatment (hypoxia, leptin) have been evaluated with respect to their own control MEAs.

**Figure 3:**
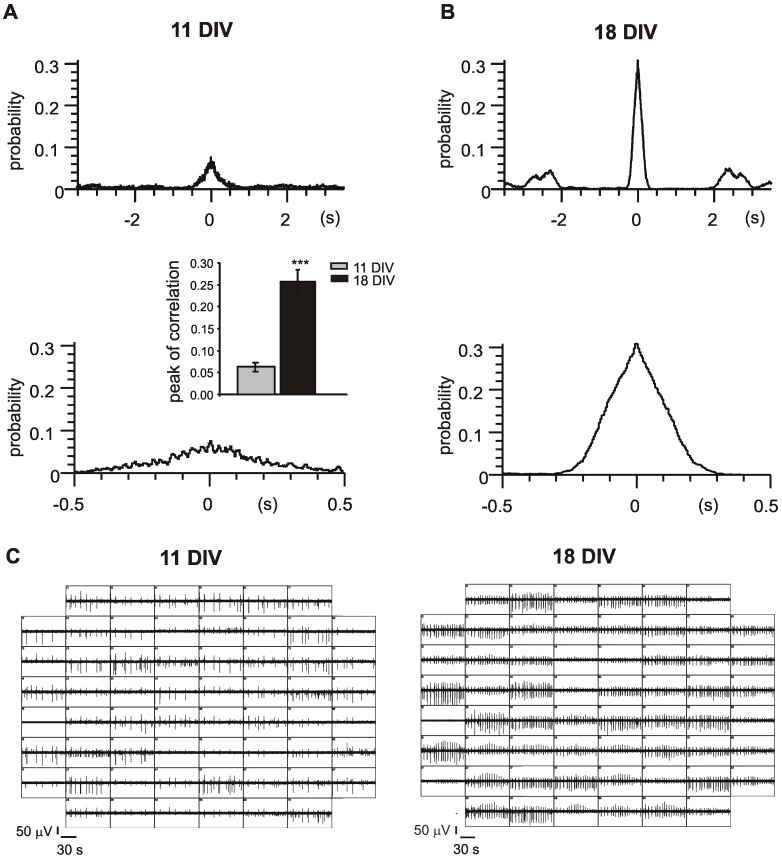
Synchronization of the activity among the electrodes increases with time in culture. Probability vs time diagrams indicating the cross-correlation between one reference electrode and another representative channel, respectively at DIV 11 (**A**) and DIV 18 (**B**). The diagrams are plotted at two different time scales: ±3.5 s (top) and ±0.5 s (bottom). Inset shows the net increment of cross-correlation between the electrodes after 18 DIV (black bars) in comparison with younger cultures (light grey bars) (p<0.001). Data are expressed as means ± S.E.M and statistical significance was calculated by using Student’s paired t-test. Values of p<0.05 were considered significant. **C**. Different synchronization on DIV 11 and DIV 18 is shown for two representative MEAs.

### Analysis of MEA Activity

Bursts analysis was performed using Neuroexplorer software (Nex Technologies, Littleton, MA, USA) after spike sorting operations. A burst consists of a group of spikes with decreasing amplitude [20], thus we set a threshold of at least 3 spikes and a minimum burst duration of 10 ms. We set interval algorithm specifications such as maximum interval to start burst (0.17 sec) and maximum interval to end burst (0.3 sec) recorded in 0.02 s bins.

We performed the burst analysis using two different approaches, either by considering the contribution of each recording channel or by calculating for each MEA one mean value over the active recording channels; we obtained comparable results of the spike parameters, suggesting a reliable homogeneity of data.

Burst analysis has been performed by monitoring the following parameters: number of spikes, frequency, number of bursts and percentage of spikes in bursts.

Cross-correlation probability vs. time diagrams were constructed by means of Neuroexplorer software (Nex Technologies, Littleton, MA, USA), using ±0.5 s and ±3.5 s and 5 ms bin size.

Data are expressed as means ± S.E.M and statistical significance was calculated either by using Student’s paired t-test or with a one way ANOVA followed by a Bonferroni post-hoc analysis. Values of p<0.05 were considered significant.

Figures were edited by means of Corel Draw Graphics Suite 12 (Corel Corporation, Ottawa, Canada).

**Figure 4:**
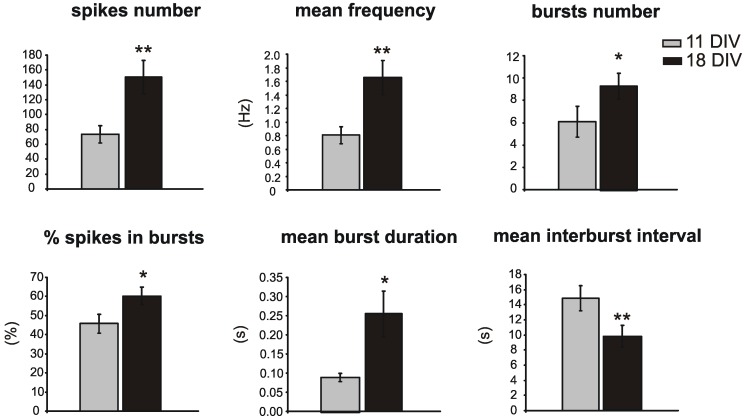
Firing frequency and bursts number increment with time in culture. Parameters characterizing electrical spontaneous activity of the hippocampal culture at DIV 11 (n = 29 MEAs; grey bars) and at DIV 18 (n = 36 MEAs; black bars) given as mean ± S.E.M. The difference between the two groups is statistically significant for all parameters (* p<0.05, **p<0.01 and ***p<0.001; using Student’s paired t-test). Spikes and bursts numbers both increased between 11 and 18 DIV. Number of spikes increased from 74±11 to 150±23 (p<0.01 ), while the number of bursts from 6.1±1.4 to 9.4±1.2 (p<0.05). The Maturation of the culture led to a prolongation of bursts duration (from 0.09±0.01 s at 11 DIV to 0.26±0.06 s at 18 DIV; p<0.02) and an increment of the percentage of spikes in the burst (46±5% vs 60±5%; p<0.03). Prolonged bursts and higher frequency on 18 DIV were accompanied by decreased mean interburst interval (IBI). Young cultures presented a mean IBI value of 15±2 s while, in mature cells (18 DIV), this interval was reduced to 9.7±1.4 s (p<0.01).

### Cell Viability Assay

Cell viability was assessed using the Trypan blue exclusion assay and plating hippocampal neurons on plastic dishes (IBD, Germany) patterned with a grid of 49 square areas of 500×500 µm^2^. Neuronal cultures were stained with 0.4% Trypan blue (Sigma-Aldrich, St. Louis, MO). Cells that were stained blue were considered dead cells and not included in the cell count, while those with a translucent clear appearance were considered alive and, therefore, included in the cell count. Neurons were counted via a light microscope under×20 magnification; ten non-overlapping fields of four different dishes were analyzed, considering the same fields of the dish before and after hypoxia (2 h 3% O2). This assay was performed either on 11 DIV (four dishes) or on 18 DIV neurons (four dishes).

## Results

### Synchronization and Firing Frequency of Hippocampal Neurons Increased with Days in Culture

Spontaneous firing of hippocampal neurons was monitored for up to three weeks after plating. We tested 72 MEAs in our experiments, and we found that hippocampal neurons became spontaneously active at DIV 8 (days in vitro). From the analysis we discarded only 10% of the arrays, in which the majority of channels (75% or more) remained silent. In addition, we selected DIV 11 as the day for monitoring the spontaneous firing of younger neurons, and we compared their activity with those of 18 DIV neurons, to assess the *in vitro* functional changes of the network activity during development. Figure 1 shows two representative photographs of the same hippocampal culture at the two stages of development (11 and 18 DIV).

Firing properties of hippocampal neurons significantly varied with time: spontaneous activity was mainly characterized by isolated spikes with limited number of bursts at DIV 11 (Fig. 1A). Neurons spontaneously fired trains of single spikes with the typical shape of extracellularly recorded action potentials (APs), i.e., a large and fast inward deflection followed by a small and slow outward deflection lasting few milliseconds (see inset) that correspond to the negative first derivative of intracellularly recorded APs [19], [21]–[23] One week later (18 DIV, Fig. 1 B), the firing changed significantly. There was a visible increase of spiking frequency, as well as a prevalence of synchronized bursts of APs (Fig. 1 B, inset) that lasted tens of milliseconds and were rarely present in younger cultures. This effect is more evident when comparing the raster plots of Fig. 2, where the APs monitored by each active recording channel are translated in a sequence of brief vertical lines versus time. Neuronal activity is distributed more or less randomly over time in younger cultures, whereas becomes more synchronized at DIV 18, when nearly all recording channels display synchronous AP bursts for tens of seconds (rectangle in Fig. 2).

To better quantify the occurrence of synchronized activity with increasing age of the culture, we measured the probability of coincidence of the single events between different electrodes [24], [25]. As shown in the cross-correlograms of Fig. 3, calculated at different time windows (±0.5 s and ±3.5 s with 5 ms bin size), the maximal correlation of neuronal activities at t = 0 s is increased by more than 300% at DIV 18 (from 0.06±0.01 to 0.26±0.03; p<0.001; see inset in Fig. 3). This indicates that synchronization of APs activity in mouse hippocampal network is greatly enhanced during neuronal maturation in culture. In this regard, representative recordings from the same MEA are shown in Fig. 3C.

**Figure 5:**
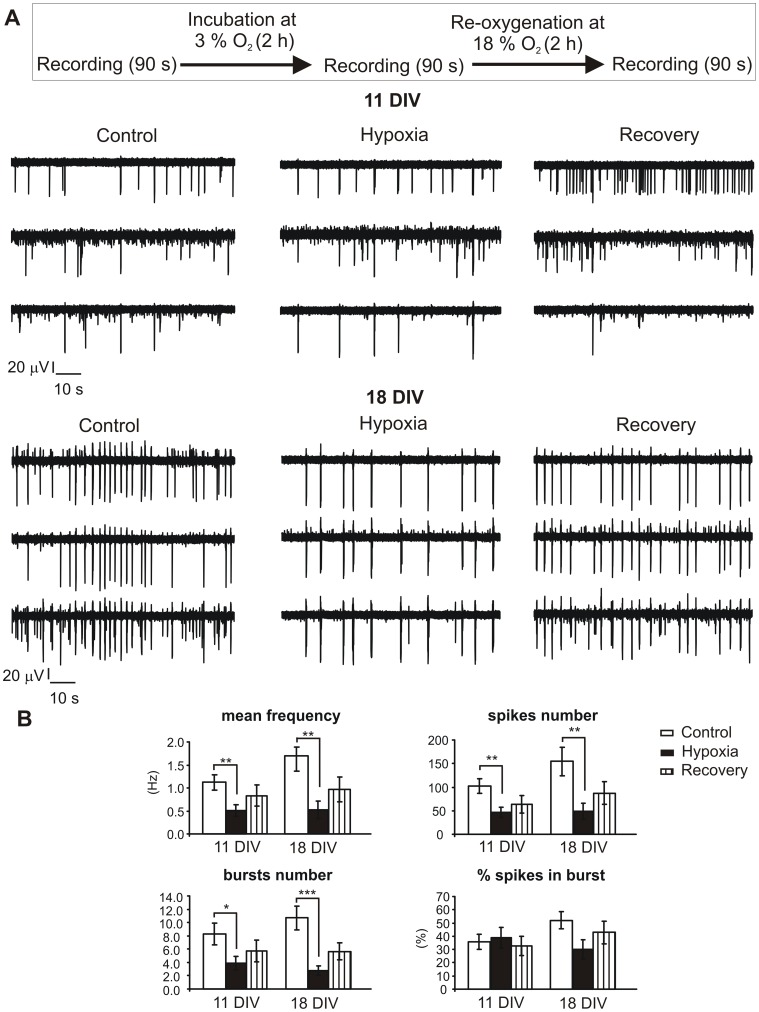
A period of hypoxia reduces spontaneous electrical activity in hippocampal cultures. **A.** Outline of the experimental protocol used to study the effect of hypoxia on hippocampal primary cultures (top). Below some examples of recordings from two representative MEAs in control conditions, 10 minutes after hypoxia and 2 hours after the treatment, at 11 and 18 DIV. It is clearly visible the reduction of the firing frequency after the exposure to lower oxygen supply (3% of oxygen for 2 hours). **B.** Main parameters characterizing electrical spontaneous activity of the hippocampal culture at 11 DIV and 18 DIV (n = 15 and n = 16 MEAs, respectively) in control conditions (white bars), after hypoxia (black bars) and 2 hours after the post-hypoxic recording (striped bars). For 18 DIV neurons, firing frequency decreased from to 1.7±0.3 Hz to 0.5±0.2 Hz (p<0.01) after hypoxia. Number of bursts and number of spikes were respectively reduced from 10.7±1.8 to 2.8±0.7 (p<0.001) and from 155±30 to 48±17 (p<0.01). In 11 DIV firing frequency decreased from to 1.1±0.2 Hz to 0.51±0.12 Hz (p<0.01) after hypoxia. Number of bursts and number of spikes were similarly reduced, the first one from 8.3±1.6 to 3.9±1.0 (p<0.05), the second one from 102±15 to 46±11 (p<0.01). At both ages, the percentage of spikes in burst did not change after hypoxia. For all parameters, recovery was not significantly different from the hypoxic group. Data are given as mean ± S.E.M. (* p<0.05, **p<0.01 and ***p<0.001, using one way ANOVA followed by a Bonferroni post-hoc analysis).

### Firing Properties of Hippocampal Neurons Under Normoxic Conditions

Because of the different spontaneous firing activity between young and elder cultures, separated analysis at DIV 11 and DIV 18 networks were carried out. Control data were taken from recordings under normoxic conditions. As shown in Fig. 4, all the parameters characterizing the spontaneous firing were significantly different between younger and elder cultured neurons. At 18 DIV, the firing frequency almost doubled (from 0.80±0.13 to 1.7±0.3 Hz, p<0.01) and this effect was accompanied by an increased number of bursts, from 6.1±1.4 to 9.4±1.2 (p<0.05). In agreement with the observation that burst activity is the main feature at 18 DIV, the burst duration and the percentage of spikes within the burst significantly increased, whereas the mean interburst interval (IBI) was reduced at 18 DIV (see legend of Fig. 4). Tetrodotoxin (300 nM) completely blocked the spontaneous activity, both at DIV 11 and DIV 18, confirming the all-or-none nature of the APs driven by TTX-sensitive Nav1 channels (data not shown).

### Hypoxia Reduced the Firing Activity but Increased the Synchronization

Before focusing on the effects of leptin, we studied how oxygen reduction could alter the network activity, by exposing the hippocampal cultures to hypoxia (3% O_2_) for 2 h. Since hypoxic-ischemic conditions can be achieved with different incubation times in primary neuronal cultures, ranging from 2 min up to 4 h exposure to low oxygen tension, we decided to use a protocol of 2 h [26]–[29]. Hippocampal activity was monitored for 90 s under three different conditions: initially under normoxia (controls), immediately after 2 h of hypoxia (3% O_2_) and after recovery from hypoxia (Fig. 5, top).

By measuring the activity of hippocampal neurons at 18 DIV (n = 16 MEAs), we found that hypoxia reduced the mean frequency by 69% (from 1.7±0.3 Hz to 0.5±0.2 Hz, p<0.01) in the majority of electrodes (79%, Fig. 5), while leaving the remaining unaffected. This reduction was accompanied by a significant decrease of the number of bursts (from 10.7±1.8 to 2.8±0.7; p<0.001), while the percentage of spikes in the burst was not significantly altered (52±6% versus 30±7%, Fig. 5B). Hypoxia therefore limits bursts onset without significantly affecting burst properties. Two hours after the end of the hypoxic treatment, when returning to normoxic conditions, mean values of firing frequency (1.0±0.3 Hz) and burst activity (5.7±1.3 bursts), were still comparable to the hypoxic group (Fig. 5; ANOVA with Bonferroni post-hoc, p>0.05), suggesting that recovery was not complete. Though, a complete recovery from hypoxia could be obtained over a longer period (12–24 h, data not shown).

**Figure 6:**
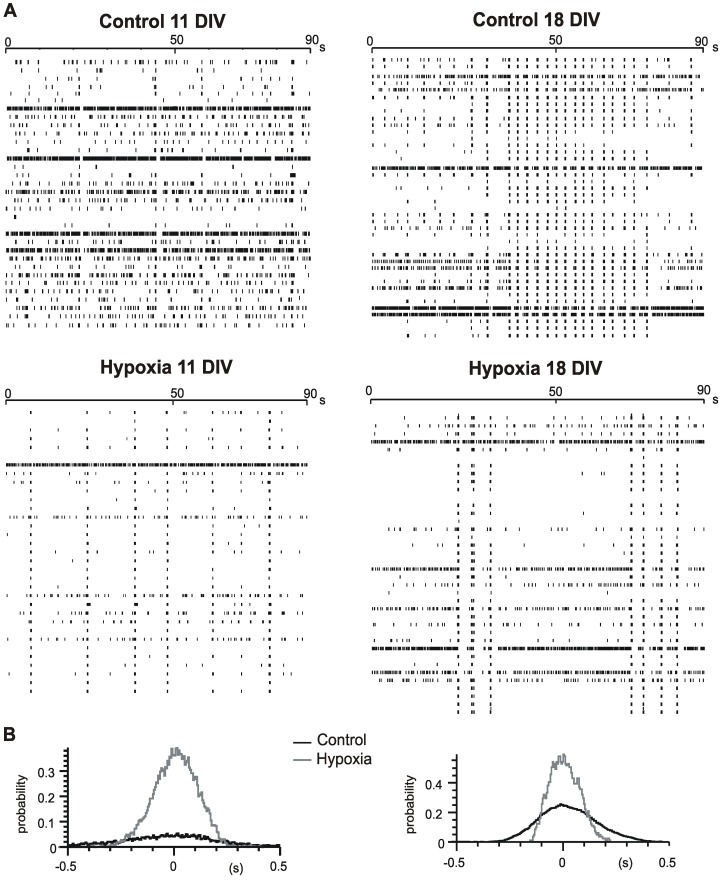
Hypoxia increases the synchronization of the spontaneous activity. **A**. Raster plots showing the activity from different channels of a MEA at 11 (left) and 18 DIV (right) in control condition and after the hypoxic treatment (bottom). **B.** Cross-correlogram plots showing the enhancement of cross-correlation after hypoxia (grey traces) vs control conditions (black traces), at 11DIV (left) and 18 DIV (right). Probability of coincidence detection increased by 600% and by 76%, respectively. In both cases, firing frequency is drastically reduced.

Concerning the pattern of electrical activity, we found that hypoxia drastically increased the occurrence of synchronized events even in younger cultures, as shown by the cross-correlogram plots in Figure 6.

When the same hypoxic treatment was performed on younger cultures (11 DIV, n = 15 MEAs), where spontaneous firing occurs through the generation of single spikes rather than synchronized burst activity, we again assessed that hypoxia caused significant effects in 62% of channels, where the firing frequency was reduced by 54% (from 1.1±0.2 to 0.51±0.12, p<0.01). Number of spikes and number of bursts underwent a similar reduction: the former decreased from 102±15 to 46±11 and the latter from 8.3±1.6 to 3.9±1.0 after hypoxia. Similarly to the elder group, recovery after hypoxia was not complete within 2 hours. Remaining electrodes (38%) showed no significant changes.

Our data indicate that hypoxia indeed reduces the firing frequency of hippocampal neurons, but at higher degree in elder cultures. Such variability of responses can be attributed to the heterogeneity of the culture, where the mixture of pyramidal neurons and interneurons may be differently firing, or else to the developmental changes of cultured neuronal networks occurring in vitro [24].

**Figure 7:**
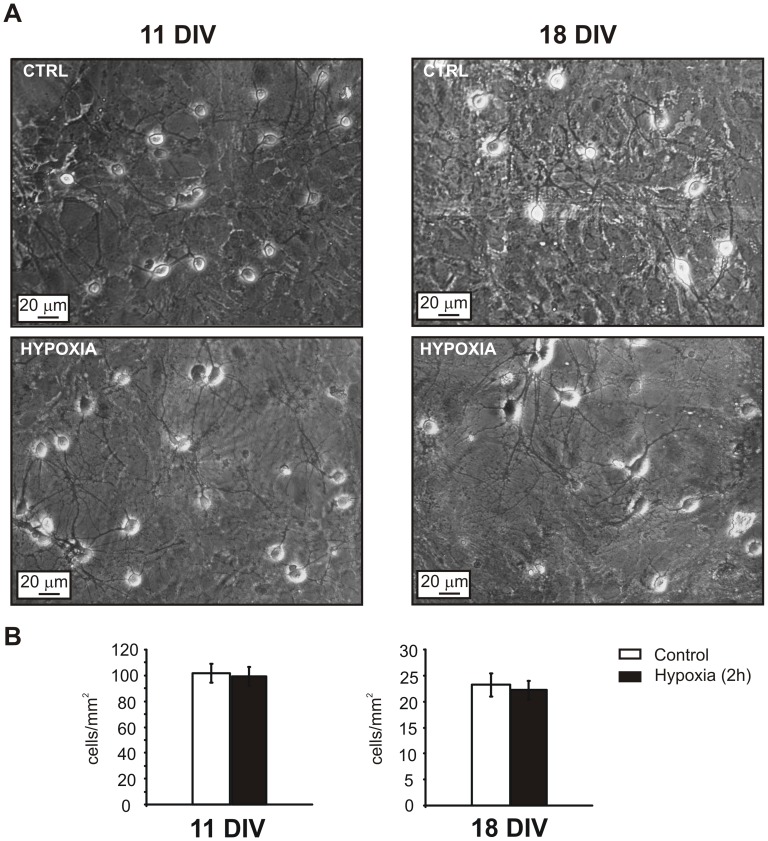
Exposure to 2 hours of hypoxia does not affect the viability of hippocampal neurons. **A.** Images of the mouse hippocampal primary culture in control conditions (top) and after 2 hours exposure to 3% O_2_ (bottom), at 11 (left) and 18 DIV (right). Cell bodies, axons and dendrites morphology were not affected by the reduction of oxygen supply. **B.** Bar histogram showing the mean number of cells/mm^2^, counted before and after the hypoxic treatment by the trypan blue exclusion assay. The number of vital cells did not significantly change after hypoxia, either at 11 DIV (left) or 18 DIV (right) (n = 4 dishes, p>0.05, using Student’s paired t-test).

In order to assess if the altered firing activity induced by hypoxia was due to cell death, we performed the trypan blue exclusion assay (see Material and Methods). This organic dye selectively stains dead/dying cells. Under control conditions, the density of unstained living cells at 11 DIV was approximately 102±7 cells/mm^2^ and remained unaltered after the hypoxic treatment (99±7 cells/mm^2^, n = 4 dishes, p>0.05 Student’s paired t-test, Fig. 7B). Concerning the effect of hypoxia on cell density on 18 DIV cultures, again, we did not reveal any statistical significant reduction of this parameter (23±2 vs 22±2 cells/mm^2^, n = 4 dishes, p>0.05 Student’s paired t-test, Fig. 7B). Moreover, looking at the morphology of the culture, cell bodies and dendritic trees were not affected by the treatment (Fig. 7A). These data indicate that cell density and viability were not affected by hypoxia.

**Figure 8:**
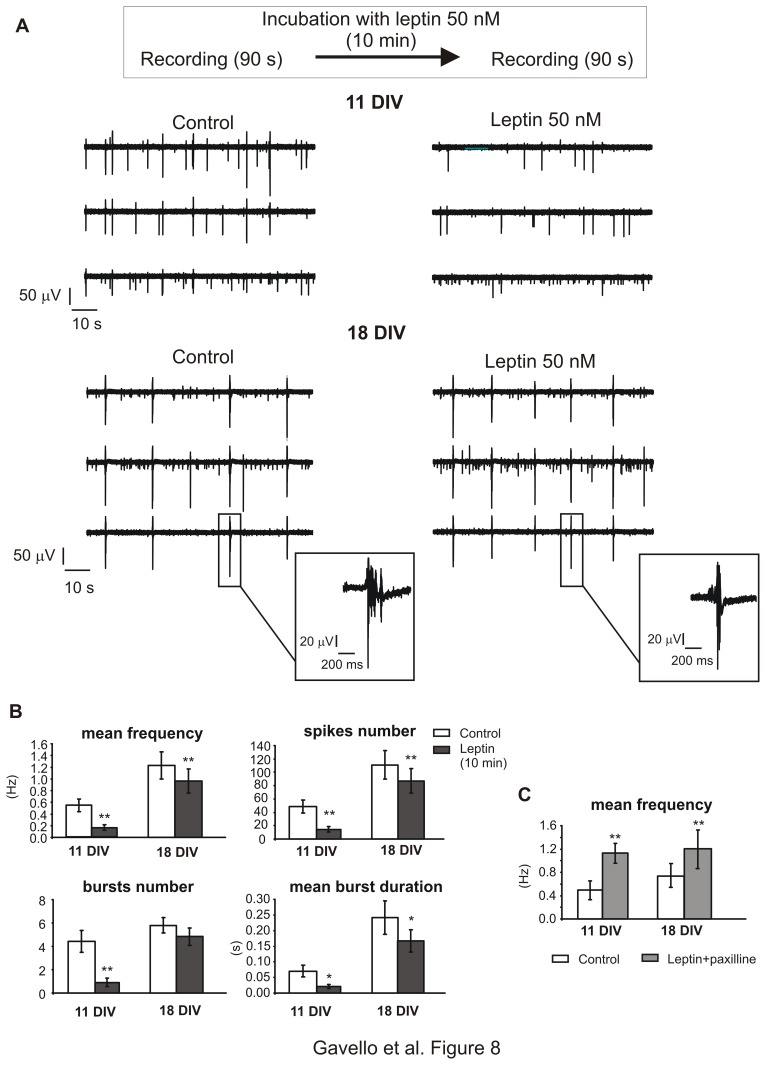
Leptin reduces firing activity in hippocampal cultures at any age. **A.** Top. Experimental protocol used to study the effect of leptin (10 minutes incubation) on hippocampal primary cultures. **Below**: recordings from two representative MEAs in control conditions and after application of leptin (10 min), at 11 or 18 DIV. The insets show a magnification of a single burst: it is clearly visible the reduction of bursts duration after leptin’s treatment at 18 DIV. **B.** Mean frequency, spikes number, bursts number and bursts duration, in control conditions (white bars) and after 10 minutes application of leptin (grey bars). Data averaged from n = 7 and n = 10 MEAs, respectively, for 11 DIV and 18 DIV cultures. Data are given as mean ± S.E.M. (* p<0.05 and **p<0.01; using Student’s paired t-test). **C.** Mean firing frequency measured in control conditions (white bars) and after addition of leptin 50 nM and paxilline 1 µM to the culture medium (light grey bars). At both ages, paxilline together with leptin significantly potentiated the mean frequency with respect to controls in approximately half of the electrodes (n = 8 MEAs for 11 DIV and n = 7 for MEAs 18 DIV; p<0.01 using Student’s paired t-test). In the remaining, paxilline plus leptin either had no effect (18 DIV) or strongly limited the reduction (see text).

**Figure 9:**
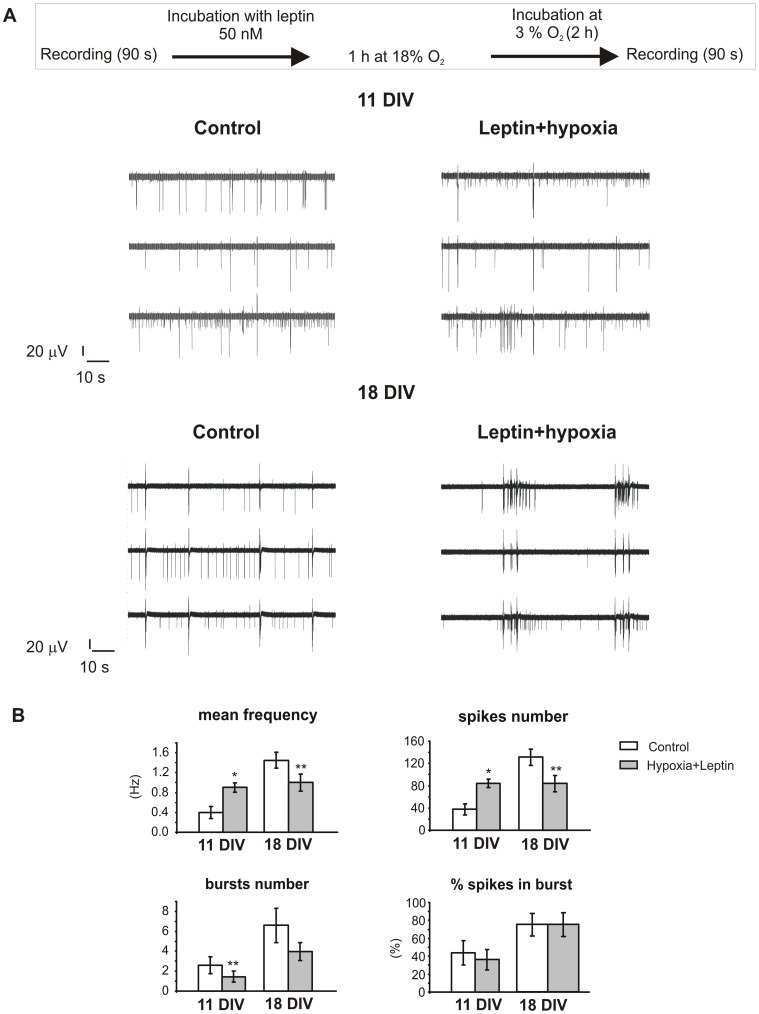
Pre-treatment with leptin partially retrieves the depressive action of hypoxia. **A.** Outline of the experimental protocol used to study the effect of leptin pre-treatment before exposure to hypoxic injury (top). Below: representative recordings in control condition and after hypoxic treatment in presence of leptin (11 and 18 DIV). The reduction of the firing frequency is still present but not so marked as with hypoxia. **B.** Main parameters characterizing electrical spontaneous activity of the hippocampal culture at 11 DIV and 18 DIV (n = 9 and n = 6 MEAs, respectively) in control conditions (white bars) and after pre-treatment with leptin before hypoxia (light grey bars). Regarding 18 DIV neurons, firing frequency decreased from to 1.5±0.2 Hz to 1.0±0.2 Hz (n = 6, p<0.01) after hypoxia. The number of spikes was reduced from 131±15 to 84±15 (p<0.01). On the contrary, the number of bursts and the percentage of spikes in bursts were not significantly reduced by the hypoxic treatment. In 11 DIV cultures, firing frequency and number of spikes were both increased after hypoxia: the former from 0.40±0.09 Hz to 0.90±0.13 Hz (n = 9; p<0.05), the latter from 38±8 to 84±11 (n = 9, p<0.05). The number of bursts was reduced from 2.6±0.9 to 1.5±0.5 (p<0.01). The percentage of spikes in bursts was not significantly altered by hypoxia. Data are given as mean ± S.E.M. (* p<0.05, **p<0.01 and ***p<0.001; using Student’s paired t-test).

**Figure 10:**
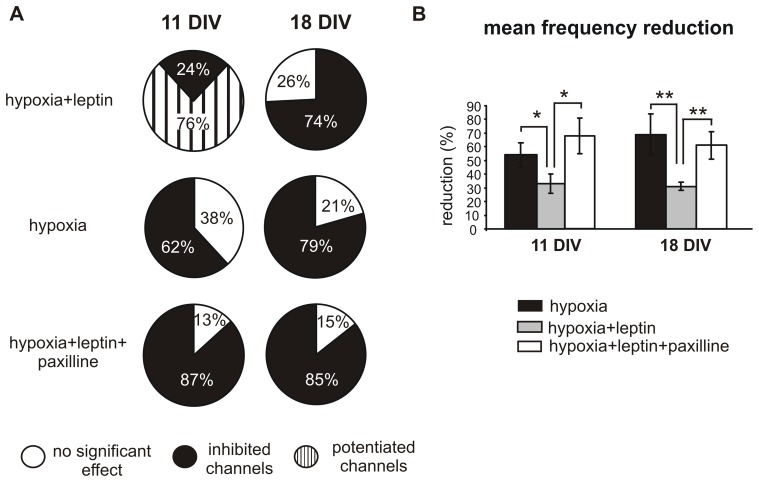
Leptin counteracts the effect of hypoxia on hippocampal neurons. **A.** Summarizing pie charts showing the percentage of electrodes potentiated (striped area), inhibited (black area) and unaltered (white area) by hypoxia, hypoxia+leptin and hypoxia+leptin+paxilline, either at 11 or 18 DIV. **B. Top.** Percentage of firing frequency reduction with respect to controls under the indicated conditions: hypoxia (black bars), leptin + hypoxia (light grey bars), leptin+hypoxia+paxilline (white bars). Leptin lowered the firing frequency reduction, either at 11 or 18 DIV (p<0.05 and p<0.01 respectively), while the addition of paxilline removed this effect.

**Figure 11:**
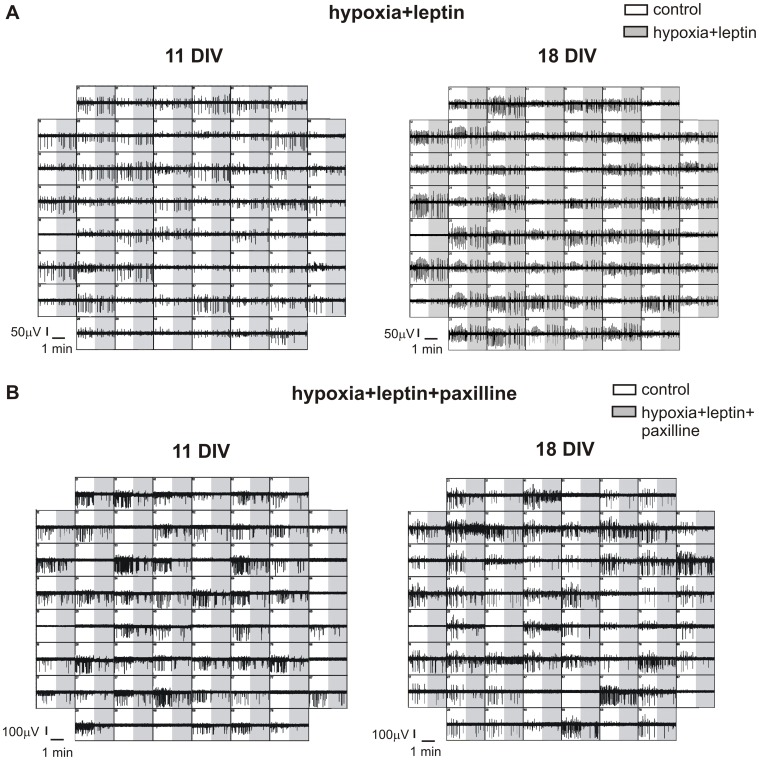
Leptin opposes to the depressive action of hypoxia through BK channels. **A.** Representative MEAs showing hippocampal spontaneous activity monitored for 90 seconds in normoxia and for 90 seconds after hypoxia+leptin treatment (light grey area). **B.** Representative MEAs showing hippocampal spontaneous activity monitored for 90 seconds in normoxia and for 90 seconds after hypoxia+leptin+paxilline treatment (light grey area). The hypoxic-induced inhibition of the firing rate is restored when leptin is applied together with paxilline.

### Leptin Reduced Spontaneous Activity of Normoxic Hippocampal Neurons

A preliminary series of experiments was devoted to investigate the activity of the neuronal network after 10 min application of 50 nM leptin, and keeping the adipokine in the culture medium also during the recordings [5], [2], [30]. These experiments were performed under normoxic conditions. On average, leptin reduced the firing frequency both in younger and elder cultures, although the effect was more pronounced on 11 DIV neurons.

More specifically, on DIV 11 (n = 7 MEAs), leptin markedly reduced the firing frequency by 71% with respect to controls (from 0.54±0.11 Hz to 0.16±0.04 Hz; p<0.01). A similar reduction has been observed also for the mean burst duration (70%) and the number of bursts, which underwent 79% decrease after the treatment (Fig. 8B). This reduction of the spontaneous activity was present in most of the microelectrodes (72%), whereas, in the remaining, electrical activity was not affected by leptin. When the BK channel blocker paxilline (1 µM) was added, the leptin-mediated reduction was either drastically lowered (from 71% to 21%), or even completely removed in 45% of cases, where the firing frequency was potentiated (from 0.5±0.2 Hz to 1.1±0.2, p<0.01), suggesting a BK-channel mediated action of leptin on normoxic neurons [5]. This opposite modulation of the firing frequency, which can be inhibited by leptin or even potentiated by leptin plus paxilline, is shown in Fig. 8B and 8C, respectively.

Regarding 18 DIV neurons, the effects of leptin were milder: spontaneous activity was reduced only by 22% (from 1.2±0.2 Hz to 1.0±0.2 Hz; p<0.01) and in a minor percentage of cases (55% versus 72%), the remaining being unaffected. Addition of paxilline together with leptin, not only removed this inhibition, but uniquely led to an increased firing frequency in 50% of the channels (from 0.74±0.19 Hz to 1.2±0.3 Hz; p<0.01, Fig. 8C), suggesting that, also in elder cultures, when BK channels are blocked, leptin action is reversed. In the remaining electrodes no significant effects were caused by adding paxilline to leptin.

The effects of leptin (50 nM) were also evaluated after 3 hours of application, in order to assess if prolonged exposures could lead to different effects on hippocampal excitability. We found that spontaneous electrical activity was reduced by leptin independent of the duration of the treatment, at both ages. On DIV 11 (n = 6 MEAs), leptin reduced the firing frequency (from 0.90±0.09 Hz to 0.48±0.13 Hz; p<0.001) in 60% of the electrodes, without significant effects on the remaining. This behaviour persisted also at DIV 18 (n = 8), where 64% of the electrodes showed a reduction of firing frequency, from 1.1±0.2 Hz to 0.76±0.13 Hz, (p<0.001), while the remaining were unaffected.

Our findings on the reduced firing frequency and burst duration by leptin are in good agreement with recent data, showing that in rat hippocampal cultures and slices leptin has a partial inhibitory action which is ascribed to a PI3-kinase-dependent activation of BK channels [30], [2].

Activation of BK channels is also in line with the reduction of bursts duration that we measured after leptin treatment. Block of BK channels in fact induces prolonged bursts in hippocampal neurons [31]. Taken together, our data support a role of BK channels as one of the molecular targets of leptins action.

### Leptin Reverses the Hypoxic Inhibition through the Activation of BK Channels

In order to assess whether leptin (50 nM) could limit the hypoxic-induced effects, we applied the adipokine 1 h before exposing the hippocampal culture to 3% of O_2_ [8] and maintained the same leptin concentration during the hypoxic treatment (Fig. 9A). Under these conditions, on 18 DIV (n = 6 MEAs), hypoxia still inhibited the firing frequency compared to controls (Fig. 10A), as shown in Figure 9A. Interestingly, this reduction occurred with a drastically lower extent than the one observed without leptin (31% versus 69%, p<0.05, Fig. 10B). Furthermore, pre-treatment with leptin preserved the bursts activity in elder cultures, thus completely reversing the effects of hypoxia on the number of bursts and the number of spikes in the burst.

The effects of leptin on DIV 11 hypoxic neurons (n = 9 MEAs) were significantly different with respect to elder cultures. Firing frequency was potentiated in 76% of cases (Fig. 10A), with more than a two-fold increase versus controls (from 0.40±0.09 Hz to 0.90±0.13 Hz; p<0.05; Fig. 9B). In the remaining we found that, similarly to the elder group, hypoxia still caused a reduction of the firing frequency (from 0.44±0.12 to 0.29±0.09 Hz), even if significantly lower than the one in the absence of leptin (33±7% versus 54±9%, p<0.05, Fig. 10B). Representative traces of this dual pattern of activity are shown in Figure 9A, where the firing frequency decreases in the upper trace while increases in the remaining.

Taken together these findings suggest that the overall action of leptin is to reverse the inhibitory effects of hypoxia on spontaneous firing, even though with more pronounced effects on younger versus elder cultures. Although in the presence of leptin a slowdown of firing persisted during hypoxia in elder cultures, the percentage of reduction was significantly attenuated as compared to the leptin-free hypoxic group.

Interestingly, when paxilline was applied together with leptin, the inhibitory effects of hypoxia were completely restored, as the firing frequency decreased by 61±10% (18 DIV) or by 68±13% (11 DIV) with respect to controls. This pattern of activity, characterized by a strong reduction of spontaneous activity was present in the majority of electrodes (85% at 18 DIV and 87% in younger cultures, Fig. 10A).

The counteracting effects of leptin versus hypoxia and the role of BK channels in the leptin-mediated pathway are summarized respectively in Fig. 11 A and Fig. 11 B. The presence of leptin during hypoxia restores the firing frequency observed under control conditions, at both ages. When comparing the firing frequency of controls (white areas) versus leptin+hypoxia (grey areas), channel activity remains unaltered, as clearly visible in Figure 11 A. On the contrary, when paxilline is added together with leptin on hypoxic neurons (Fig. 11 B), firing frequency is severely impaired: spontaneous activity of hippocampal neurons is reduced to a comparable extent occurring during hypoxia alone (Fig. 5).

Overall these data suggest that: *i)* leptin opposes to the hypoxia-induced inhibition, and *ii)* leptin-mediated pathway involves the activation of paxilline-sensitive BK channels.

## Discussion

Using the MEAs, we have provided evidence that chronic hypoxia affects neuronal excitability of mouse hippocampal primary cultures, mainly reducing the firing rate of DIV 11 and DIV 18 neurons and increasing their synchronized firing. Moreover, we investigated from a functional point of view the effects of leptin, demonstrating that leptin pre-treatment successfully opposes the depressive effects induced by hypoxic injury on hippocampal excitability. These findings may be associated to the functional impairment of central neuronal populations occurring at the first symptoms of neurodegenerative disorders, and thus address to the potential neuroprotective role of leptin.

### Network Burst Activity Under Normoxia

Our data clearly show that hippocampal network activity changes along with maturation, switching from single spikes to synchronized bursts. This pattern of activity, also present in many brain areas such as cortex [32] and midbrain dopaminergic neurons [33], has been matter of debate for a long time and seems to derive from the balance between neuronal excitation and synaptic inhibition, rather than being the consequence of strong depolarization [20]. During a burst, the amplitude of APs is typically reduced, due to a balance between excitation and inhibition [34], while burst duration is regulated by vesicle pools availability [35].

We pointed out that: *i)* network synchronization is tightly correlated to the development of the network, as indicated by the net increment of the cross-correlation index between the recording electrodes at DIV 18, probably ascribed to the increased number of connections between neurons in culture [14], and *ii)* bursts are the main feature of spontaneous activity in mature hippocampal cultures, in agreement with the fact that bursts are triggered when neuronal spike activity is quicker, during mature stages of neuronal networks development [34]. These changes in the firing patterns of hippocampal networks are relevant for a variety of physiological mechanisms, like synaptic plasticity and circuits development [36]. Furthermore, bursts synchronization in neuronal networks can be promoted by learning, as shown by Li et al. [25] taking advantage of the MEA technology.

### Hypoxia Decreases the Firing Frequency and Synchronizes the Events

Our data on hippocampal neuronal activity demonstrate that *chronic* oxygen reduction significantly decreased both firing frequency and burst activity at any age (11 and 18 DIV), with more pronounced in elder cultures. Even though patch-clamp studies would be required for investigating the molecular basis of this process, our findings are in good agreement with the observation that, in response to oxygen deprivation, rat CA1 pyramidal neurons exhibit an hyperpolarization (hypoxic hyperpolarization), associated with a reduction of cell input resistance [37]. This hyperpolarization is mediated by the activation of both ATP-sensitive and Ca^2+^-dependent K^+^ channels, due to the rise of Ca^2+^ released from intracellular stores [37]. These findings could thus explain the reduced firing frequency and burst activity recorded in our experiments after the hypoxic period. On the contrary, *acute* exposure to oxygen-glucose deprivated medium (OGD) produces opposite effects, by transiently increasing the firing frequency [12], up-regulating L- and N–type Ca^2+^ channels in CA1 hippocampal neurons [38], or activating a TRPM7 channel, permeable to Ca^2+^ and Mg^2+^ ions [26]. It is worth noticing that this initial up-regulation of the firing frequency, which occurs following *acute* instead of *chronic* hypoxia, might downstream activate Ca^2+^-dependent K^+^ channels, thus reducing the firing frequency on a longer time scale, as we observed in hippocampal neurons after 2 h hypoxia. Moreover, we show that hypoxia increases the synchronization of neuronal spontaneous activity either at 11 or 18 DIV. The alteration of the functional network connectivity after hypoxia was previously reported by Galan et al. [39], and it is independent of changes in the firing rate.

Thus we provide here a functional evaluation of spontaneous firing impairment in hippocampal primary cultures, following chronic hypoxia, by means of the MEA technology. This issue is not deeply explored in literature, since only few reports show the effect of hypoxia on neuronal activity using the microelectrode array technique [40], [12].

### Leptin Counteracts the Hypoxia-induced Inhibition of Firing Frequency through BK Channels Activation

In the last few years an increasing number of reports has pointed out the pivotal role of leptin signalling in learning, memory and neuroprotection, as its receptor is found to be expressed also in extra-hypothalamic regions such as the hippocampus [41], [42]. The discovery of leptin’s action in cognition and neuronal survival triggered a strong interest on the possible correlation between leptin levels and neurodegenerative disorders, like the Alzheimer disease (AD). Recent works have indentified leptin levels as good indicators of susceptibility to prevent AD in elderly population: higher plasma concentrations of leptin correlated with a significantly lower risk of dementia and AD [43]. Alzheimer disease could be defined as a brain expression of metabolic disorder, involving the disregulation of lipid metabolism and thus positively correlating with obesity [44]. Moreover, it is proved that leptin ameliorates the pathology of CRND8 transgenic mice, a model of AD, by reducing the levels of tau phosphorylation and β-amyloid *in vitro* [45], [46]. Thus, there is an increasing interest in exploring the possible use of leptin in therapeutic treatments to prevent this neurodegenerative disorder.

A role of leptin as neuroprotective agent of hypoxic-ischemic brain injury is highlighted in recent works, mostly focusing on cell survival and intracellular pathways [47]. Leptin protects hippocampal neurons against cell death, induced by excitotoxic and oxidative insults, by activating a PI3-kinase-mediated pro-survival signalling pathway, which involves the activation and phosphorylation of Akt [28], [48].

These issues, concerning the role of leptin against hypoxic injury, have been investigated here for the first time by analyzing the functional properties of hippocampal networks. Our main finding is that leptin differently acts on hippocampal neurons during development. Leptin approximately halves the hypoxia-induced inhibition of the firing frequency on elder neurons (DIV 18). Leptin is supposed to exert its effect through large-conductance BK channels, since the addition of paxilline causes the hypoxic inhibition to be completely restored. In this respect, patch-clamp experiments will be useful to assess whether leptin effectively hyperpolarizes the membrane resting potential against a prolonged depolarization evoked during hypoxia [2], [5], [30]. A prominent role of BK channels in neuroprotection is supported indeed by a number of works on transient cerebral ischemia, which reveal the involvement of BK channels in promoting neuronal survival [49], [50]. As proposed by Hu [51], activation of BK channels may serve as an “emergency brake”, preventing cell damage or apoptosis under pathophysiological conditions that result in a large Ca^2+^ transient, such as hypoxic/ischemic injury. Thus, the activation of BK channels by leptin could counteract the hypoxic depolarization [12], [52], repolarizing the cells, so protecting the neurons from the post-hypoxic reduction of firing frequency. In accordance with this concept, BK channel openers are reported to provide significant cortical neuroprotection during acute brain ischemia [53].

Still more pronounced effects, induced by leptin, have been found on DIV 11 hippocampal neurons, since leptin completely reverses the effects of hypoxia even by potentiating their firing activity. On average, leptin counteracts hypoxia on younger neurons both by reducing the number of inhibited channels and by inducing a potentiation of the firing frequency in 76% of the channels. Similarly to elder neurons, the addition of paxilline causes leptin to become ineffective against hypoxia, thus restoring the inhibition of spontaneous activity.

Thus, from a functional point of view, we can conclude that leptin acts by effectively counteracting the depressive effects of chronic hypoxia on neuronal activity. Future works will be addressed to reveal whether leptin may preferentially affect specific neuronal populations.
